# Is gentrification all bad? Positive association between gentrification and individual’s perceived neighborhood collective efficacy in Montreal, Canada

**DOI:** 10.1186/s12942-017-0096-6

**Published:** 2017-07-14

**Authors:** Madeleine Steinmetz-Wood, Rania Wasfi, George Parker, Lisa Bornstein, Jean Caron, Yan Kestens

**Affiliations:** 1 Department of Geography, McGill University, Burnside Hall, 805 Sherbrooke St W, Montreal, QC H3A 0B9 Canada; 2 University of Montreal Hospital Research Centre (CRCHUM), CHUM - Pavilion S 850, St-Denis St., Office, Montreal, QC H2X 0A9 Canada; 3 Department of Social and Preventive Medicine, École de Santé Publique de l’Université de Montréal (ESPUM), 7101, rue du Parc, Montreal, H3N 1X9 Canada; 4 GP Rollo & Associates, Land Economists, Richmond, V7A 3A8 Canada; 5 School of Urban Planning, McGill University, 815 Sherbrooke St W, Montreal, QC H3A 0C2 Canada; 6 Douglas Mental Health University Institute, 6875 Boulevard LaSalle, Verdun, QC H4H 1R3 Canada

**Keywords:** Gentrification, Collective efficacy, Epidemiological catchment area, Health

## Abstract

**Background:**

Collective efficacy has been associated with many health benefits at the neighborhood level. Therefore, understanding why some communities have greater collective efficacy than others is important from a public health perspective. This study examined the relationship between gentrification and collective efficacy, in Montreal Canada.

**Methods:**

A gentrification index was created using tract level median household income, proportion of the population with a bachelor’s degree, average rent, proportion of the population with low income, and proportion of the population aged 30–44. Multilevel linear regression analyses were conducted to measure the association between gentrification and individual level collective efficacy.

**Results:**

Gentrification was positively associated with collective efficacy. Gentrifiers (individuals moving into gentrifying neighborhoods) had higher collective efficacy than individuals that lived in a neighborhood that did not gentrify. Perceptions of collective efficacy of the original residents of gentrifying neighborhoods were not significantly different from the perceptions of neighborhood collective efficacy of gentrifiers.

**Conclusions:**

Our results indicate that gentrification was positively associated with perceived collective efficacy. This implies that gentrification could have beneficial health effects for individuals living in gentrifying neighborhoods.

## Background

Collective efficacy is a form of social capital that can be defined as the consolidation of neighborhood social cohesion and informal social control. Whereby, social cohesion embodies the concepts of mutual support and trust, and informal social control refers to the collective capacity of community members to coordinate their members to achieve collective goals according to a set of principles [1, 2]. At the community level, collective efficacy is hypothesized to influence health by means of informal social control over deviant behaviors and by means of collective actions on the part of residents advocating for the best interests of the community (ex: protests to dispose of physical hazards or to avoid cuts to community services) [3].

Collective efficacy has been associated with many health benefits at the neighborhood level such as lower rates of cardiovascular disease [4], obesity [5], sexually transmitted diseases [6], mental health outcomes [7], and all-cause mortality, [4] as well as positive perceptions of self-rated health [8]. High neighborhood collective efficacy has also been associated with decreased risky sexual behavior among youth [9] and has been shown to enhance the protective effect of family attachment and support on youth’s suicidal behaviors [10]. Neighborhoods with high levels of collective efficacy are also more likely to solicit internal and external resources to address neighborhood sources of violence, disorder and neighborhood physical hazards [8]. Given the benefits of collective efficacy to a community, gaining an understanding of why some communities have greater collective efficacy than others is important from a public health perspective. To this end, researchers have attempted to understand the neighborhood characteristics and processes that could influence collective efficacy such as built environment design [11], neighborhood attachment [12], poverty [13], and safety [13].

The pathways linking aspects of the neighborhood social and physical environment to collective efficacy can be explained by the existence of a dynamic relationship existing between humans, and their physical and social environments. Although, individuals play an important role in shaping the environment, aspects of the physical and social environment may also in turn contribute to individuals’ social and health outcomes [11]. It follows that characteristics of neighborhood built and social environments may explain the unequal distribution of collective efficacy across communities.

Gentrification is a neighborhood process that may contribute to the disruption or development of neighborhood collective efficacy, as gentrification is characterized by a rapid change in the social status and economic characteristics of a neighborhood as compared to the rest of the city [14]. It can be understood as an in-migration of higher-socioeconomic status individuals into neighborhoods of lower socioeconomic status [14–16] resulting in investments in the built environment [17], subsequent increases in property values and rents [14, 18] and is generally characterized by an upward transition in status, class, and income of neighborhood residents [18]. In some instances, gentrification may also be the result of real estate or urban developments that result in increasing land values and subsequent displacement of low socioeconomic status individuals [19]. Perspectives on how gentrification could influence collective efficacy are conflicting. At one end, theories of social mix such as Wilson 1987s social isolation thesis [20] suggest that gentrification will improve neighborhood collective efficacy, as incoming high socioeconomic status residents will encourage the propagation of values such as maintaining properties [21], have increased political influence to facilitate the demand for high quality resources, [21–25] and encourage the creation of community initiatives such as “neighborhood watches” or “citizen patrols” [21]. Conversely, a significant body of literature has revealed that gentrification is negatively associated with the two components of collective efficacy, i.e., social cohesion and informal social control. This work argues that gentrification policies negatively affect vulnerable populations and alter the original social fabric of the neighborhood, promoting social polarization, social segregation and displacement [26–31]. These processes will in turn negatively affect social cohesion and informal social control [30, 32–34], thereby decreasing the likelihood that a community will engage in collective action [25, 28, 35].

### Objective

To our knowledge no studies have undergone an empirical investigation of the relationship between gentrification and neighborhood collective efficacy as a whole. We use data from the Social and Psychiatric Epidemiology Catchment Area of the South West of Montreal (ZEPSOM) (Caron, Fleury et al. 2012) to (1) examine the relationship between gentrification and perceptions of neighborhood collective efficacy and (2) examine whether the effect of gentrification on perceptions of neighborhood collective efficacy is the same for gentrifiers (individuals moving into gentrifying neighborhoods) and the original inhabitants of the neighborhood.

## Methods

### Sample

The sample was obtained from wave 1 (year 2006) of the ZEPSOM study, an ongoing longitudinal study, organized by the Canadian Institute of Health Research Team in Social and Psychiatric Epidemiology^1^ (Caron, Fleury et al. 2012). The area under study encompassed four boroughs of Montreal: Saint-Henri/Pointe St-Charles, Lachine/Dorval, LaSalle, and Verdun. The study aimed to recruit 2400 participants from the study area aged 15–65, with proportional representation with respect to geographical location, population density, and socio-economic status (educational attainment).

Trained interviewers recruited potential participants through a door-to-door campaign. The interviewers contacted the residents who had agreed to participate in the study by phone within a week of recruitment, in order to schedule a face-to-face meeting either at the participant’s home or in an office designated for that purpose at the Douglas. However, most interviews were conducted at home. The face-to-face interview was conducted once the consent form was signed and lasted approximately 1.5–3 h.

The final sample of 2433 participants represented approximately 600 participants in each borough: Saint-Henri/Pointe St-Charles (612), Lachine/Dorval (603), LaSalle (584) and Verdun (635). The cooperation rate was 48.7%. This is superior to the median rates reported in epidemiological studies of populations conducted post year 2000 (Morton, Cahill and Hartge (2006). The study sample overrepresented women (61.6%) compared to the reference population (51.7%). Contrastingly, men under the age of 45 were underrepresented. Table 1 presents sample characteristics before and after weighting the data for sex and age. The mean age was 40.73 [standard deviation (SD) = 14.09] of whom 48% were men, 38% were single, 45% were married or in common law relationship, and 15% were divorced or separated. With respect to educational attainment, employment and immigration status 72% had a post-high school diploma, 79% were employed in the last 12 months, while 25% were immigrants. French was the primary language spoken by 55% of the respondents, while 21% spoke English as their primary language. 82% of the sample was Caucasian. The average personal income was CAN$ 33,19 (SD = $33,15) and the average family income was CAN$ 59,06 (SD = $49,85). Based on criteria from statistics Canada, 33.4% of the participants were considered to be a low income earner.

**Table 1:** Socio-demographic characteristics of the sample

	Unweighted total (n = 2433)	Weighted total^a^ (n = 2432.37)
Gender (%)		
Female	61.78	51.71
Male	38.22	48.29
Age (mean, SD)	41.39, 13.34	40.73, 14.09
Age (%)		
15–24	12.00	16.12
25–34	21.58	20.66
35–44	23.59	20.84
45–54	22.44	20.92
55+	20.39	21.46
Marital status (%)		
Single	36.48	37.95
Married	29.81	29.37
Separated	3.05	2.82
Common-law	15.81	15.86
Divorced	13.13	12.39
Widowed	1.73	1.61
Education (%)		
Less high school	15.30	15.99
High school	11.51	12.13
Post-high school	73.19	71.88
Immigrant (%)		
No	75.02	75.14
Yes	24.98	24.86
Primary language (%)		
English	21.90	20.59
French	54.25	55.36
English + French	6.59	6.56
Neither English nor French	17.25	17.50
Caucasian (%)		
No	18.69	18.46
Yes	81.31	81.54
Dwelling owned by a household member (%)		
No	61.47	61.15
Yes	38.53	38.85
Held a job in the past 12 months (%)		
No	22.60	21.41
Yes	77.40	78.59
Household size (mean, SD)	2.50, 1.39	2.49, 1.36
Household income (mean, SD)	$57,68, $49,72	$59,06, $49,85
Personal income (mean, SD)	$32,53, $31,20	$33,19, $33,15

^a^The data was weighted for sex and age

### Measures

#### Perception of neighborhood collective efficacy

Perception of neighborhood collective efficacy was measured using the Sense of Collective Efficacy Scale [2]. The Collective Efficacy Scale is a summary measure of social cohesion and informal social control, with five questions per component. It uses a 5-point Likert-scale, with questions on social cohesion such as: “Are people around here willing to help their neighbors?” and questions on informal social control such as “Do you think that your neighbors can be counted on to intervene if the fire station closest to their home is threatened with budget cuts?” Collective efficacy is often measured at the neighborhood level [2, 8, 36]. However, perceived collective efficacy can also be conceptualized as a cognitive orientation towards one’s environment [37]. Measured individually perceived collective efficacy helps to capture residents’ perceptions of cohesiveness and common goals [37] and can allow us to disentangle the effect of gentrification on individuals’ perceptions of context. Specifically, in this paper examining perceptions of collective efficacy at the individual level was necessary so that we could meet our second objective. This objective was to explore how the perceptions of neighborhood collective efficacy of gentrifiers might differentiate from the perceptions of neighborhood collective efficacy of individuals that had originally inhabited gentrifying neighborhoods before the beginning of the gentrification process.

#### Gentrification

Our measure of gentrification was developed based on the methodology of Grube-Cavers et al. (2014). Gentrification measures were compiled for each participant’s neighborhood using census tracts, obtained from the Canadian 1996, and 2006 Census datafiles. Indicators used to identify gentrifying tracts included: median household income, proportion of the population with a bachelor’s degree, average rent, proportion of the population with low income, and proportion of the population aged 30–44. *Z* scores for each measure in 1996 and 2006 were calculated using the Montreal census metropolitan area average and standard deviation. We first identified census tracts that had the potential to undergo gentrification by selecting tracts that had negative *Z* scores in 1996 for median household income, proportion of the population with a bachelor’s degree, and average rent and had a positive *Z* score for the proportion of population with low income. Census tracts with the potential to undergo gentrification were considered gentrified if the difference between the 2006 and 1996 *Z* scores was positive for all indicators except for the proportion of low income, which needed to be negative. A binary variable was then constructed to differentiate census tracts that underwent gentrification between 1996 and 2006 from those that did not.

We constructed a categorical variable: “type of resident”, based on the number of years a person lived in a gentrified or non-gentrified neighborhood with the following categories: mover into a gentrified neighborhood/potential gentrifier (lives in a census tract that had undergone gentrification and moved there in 1996 or later); resident whose neighborhood was gentrified (liveds in a census tract that had undergone gentrification and moved there before 1996); mover into a non-gentrified neighborhood (lives in a census tract that did not undergo gentrification and moved there in 1996 or later); and residents whose neighborhood wasn’t gentrified (lives in a census tract that did not undergo gentrification and moved there before 1996).

#### Health outcomes

Respondents were asked to rate their mental and physical health using a 5-point Likert-scale. We classified the responses into the following categories ‘good health’, ‘fair health’ and ‘poor health’ to create the variables “perceived mental health” and “perceived physical health”. Respondents were also asked to rate their satisfaction with life using a 5-point Likert-scale. The responses were classified into the following categories ‘satisfied with life’, ‘neither satisfied nor dissatisfied with life’ and ‘dissatisfied with life’.

#### Socio-demographic and neighborhood characteristics

The survey included questions on individual-level socio-demographic characteristics: sex, level of education, first language, housing tenure (renter vs. owner-occupant household), household income in 2006, and number of years that the participant lived in their place of residence. Most of these variables were unaltered. However first language was categorized into French versus other. Additionally, the continuous variable household income was positively skewed within the ECA-MSW sample group, therefore the base-10 logarithms of this variable was used for normalization purposes. A 2006 neighborhood poverty score was computed by taking the inverse of the sum of the tract *Z* scores for median household income, proportion of the population with a bachelor’s degree, and average rent and then adding the Z score for the proportion of the population with low income.

## Analysis

Multilevel linear regression analysis was performed to examine the relationship between gentrification between 1996 and 2006 and perception of neighborhood collective efficacy. In these model individuals (i) were nested within neighborhood census units (j). Equation one represents the null model with an intercept β_0_, the random effect for the neighborhood census unit u_0j_, and e_0i(j)_ the random effect for individuals i classified within j neighborhood census units.

$${\text{y}}_{{{\text{i}}\left( {\text{j}} \right)}} =\upbeta_{0} + {\text{u}}_{{0{\text{j}}}} + {\text{e}}_{{0{\text{i}}({\text{j}})}}$$

The level 2 and the level 1 control variables were then added to the model. At level one (individual level) we included the following: Gender β_1oi(j)_, age β_2oi(j)_, first language is not French β_3oi(j)_, level of education β_4oi(j)_, housing tenure β_5oi(j)_, household income in 2006 β_6oi(j)_, number of years lived in place of residence β_7oi(j)_. At the second level (neighborhood) of the model we included the following: neighborhood poverty in 2006 β_8o(j)_ and gentrification β_9o(j)_.

$${\text{y}}_{{{\text{i}}({\text{jk}})}} =\upbeta_{0} + {\upbeta}_{{1{\text{o}}\,{\text{i}}\left( {\text{j}} \right)}} +\upbeta_{{2{\text{o}}\,{\text{i}}({\text{j}})}} +\upbeta_{{3{\text{o}}\,{\text{i}}({\text{j}})}} +\upbeta_{{4{\text{o}}\,{\text{i}}({\text{j}})}} +\upbeta_{{5{\text{o}}\,{\text{i}}({\text{j}})}} +\upbeta_{{6{\text{o}}\,{\text{i}}({\text{j}})}} +\upbeta_{{7{\text{o}}\,{\text{i}}({\text{j}})}} +\upbeta_{{8{\text{o}}\,{\text{j}}}} +\upbeta_{{9\,{\text{oj}}}} + {\text{u}}_{\text{o j}} + {\text{e}}_{{{\text{o}}\,{\text{i}}({\text{j}})}}$$

In our second model, we performed the same analyses except we replaced the gentrification variable with the variable type of resident. Although collective efficacy was our main dependent variable of interest, we also performed multinomial logistic regressions to see if gentrification was related to health outcomes in our sample. To this end, we conducted some exploratory analyses to examine the relationship between gentrification and life satisfaction, self-perceived physical health, and self-perceived mental health using multinomial logistic regression models. All statistical analyses were conducted using SPSS version 20.

## Results

There were 1264 tracts that had the potential to undergo gentrification in 1996 and 353 of these tracts underwent gentrification between 1996 and 2006. Estimates from Table 2 indicate that women had lower collective efficacy than men (β = −1.08; 95% CI −1.70, −0.46, sig = 0.00), those where French was not their first language had lower collective efficacy than francophones (β = −1.09; 95% CI −1.75, −0.43, sig = 0.00) and renters had higher collective efficacy than homeowners (β = 2.62; 95% CI 1.88, 3.37, sig = 0.00). Household income was negatively associated with collective efficacy (β = −0.85; 95% CI −1.56, −0.14, sig = 0.02), whereas gentrification was significantly positively associated with collective efficacy (β = 1.54; 95% CI 0.16, 2.93, sig = 0.03).

**Table 2:** Multilevel linear regression model of the relationship between gentrification and collective efficacy adjusted for neighborhood and socio-demographic characteristics

	β	(95% CI)	*P* value
Constant	29.33	(25.50, 33.16)	0.00
Age	−0.01	(−0.04, 0.01)	0.31
Sex (ref. male)			
Female	−1.08	(−1.70, −0.46)	0.00
Language (ref. first language is French)			
First language is not French	−1.09	(−1.75, −0.43)	0.00
Education (ref. bachelor’s degree)			
High school or less	0.46	(−0.40, 1.31)	0.30
Post-secondary education of a lower level than a bachelor’s degree	0.61	(−0.13, 1.34)	0.11
Neighborhood poverty	0.09	(−0.30, 0.47)	0.66
Household income	−0.85	(−1.56, −0.14)	0.02
Tenure (ref. owner)			
Renter	2.62	(1.88, 3.37)	0.00
Number of years lived in dwelling	−0.03	(−0.07, 0.02)	0.20
Gentrification (ref. no)			
Yes	1.54	(0.16, 2.93)	0.03
Random effects			
Tract variance	2.43	(1.32, 4.50)	0.01

Significance level: P = 0.05

Estimates from Table 3 indicate that women had lower collective efficacy than men (β = −1.09; 95% CI −1.72, −0.47, sig = 0.00), those where French was not their first language had lower collective efficacy than francophones (β = −1.10; 95% CI −1.76, −0.44, sig = 0.00). Renters had higher collective efficacy than homeowners (β = 2.69; 95% CI 1.96, 3.42, sig = 0.00). Household income was significantly negatively associated with collective efficacy (β = −0.86; 95% CI −1.57, −0.15, sig = 0.02). Movers into neighborhoods that did not gentrify (β = −1.72; 95% CI −3.15, −0.29, sig = 0.02) and original residents of neighborhoods that did not gentrify (β = −1.96; 95% CI −3.54, −0.37, sig = 0.02) had lower collective efficacy than individuals that moved into gentrifying neighborhoods. Although a negative tendency can be observed, the collective efficacy of the original residents of gentrifying neighborhoods (β = −1.25; 95% CI −3.36, 0.85, sig = 0.24) was not significantly different than the collective efficacy of individuals that moved into gentrifying neighborhoods.

**Table 3:** Multilevel linear regression model for the relationship between type of resident and collective efficacy adjusted for neighborhood and socio-demographic characteristics

	β	95% CI	P Value
Constant	31.09	(27.13, 35.06)	0.00
Age	−0.02	(−0.04, 0.01)	0.18
Sex (ref. male)			
Female	−1.09	(−1.72, −0.47)	0.00
Education (ref. Bachelor’s degree)			
Highschool or less	0.44	(−0.42, 1.30)	0.31
Post-secondary education of a lower level than a bachelor’s degree	0.59	(−0.14, 1.32)	0.12
Neighborhood poverty	0.08	(−0.30, 0.47)	0.67
Type of resident (ref. Moved into a gentrifying neighborhood)			
Original resident of a gentrifying neighborhood	−1.25	(−3.36, 0.85)	0.24
Moved into a neighborhood that did not gentrify	−1.72	(−3.15, −0.29)	0.02
Original resident of a neighborhood that did not gentrify	−1.96	(−3.54, −0.37)	0.02
Household income	−0.86	(−1.57, −0.15)	0.02
First language (ref. first language is French)			
First language is not French	−1.10	(−1.76, −0.44)	0.00
Tenure (ref. owner)			
Renter	2.69	(1.96, 3.42)	0.00
Random effects			
Tract variance	2.44	(1.32, 4.51)	0.00

Significance level: P = 0.05

Gentrification was not significantly associated with self-perceived mental or physical health. With respect to life satisfaction, individuals living in gentrifying neighborhood had higher odds or reporting being satisfied with life (OR 1.91; 95% CI 1.05–3.49, sig = 0.03) and higher odds of reporting being neither satisfied nor dissatisfied with life (OR 2.69, 95% CI 1.41–5.14, sig = 0.00) than reporting being dissatisfied with life.

## Discussion

Studies have indicated that gentrification is linked to food insecurity [38], pre-term birth [39] and lower self-rated health in minorities [41]. According to the Center for Disease Control, gentrification may also limit access to affordable healthy housing, transportation choices, quality schools, bicycle and walking paths, and exercise facilities for vulnerable populations displaced by gentrification [40]. Our results imply that the effects of gentrification may not all be negative. These findings indicate that gentrification is beneficial for individuals’ perceptions of neighborhood collective efficacy. Collective efficacy has been shown to be associated with positive health outcomes at the neighborhood level such as lower rates of cardiovascular disease [4], obesity [5], sexually transmitted diseases [6], mental health outcomes, [7] and all-cause mortality [4]. This is contrary to the previous literature indicating that gentrification weakens the components of collective efficacy, social cohesion and informal social control [28, 30, 32–34]. Our results are instead in line with Wilson 1987 social isolation thesis that posits that incoming high income residents will encourage the propagation of values such as maintaining properties [21], have increased political influence to facilitate the demand for high quality resources [21–25] and encourage the creation of community initiatives such as “neighborhood watches” or “citizen patrols” [21]. Consequently, gentrification, through means such as increased social mix, will promote collective efficacy [32, 42].

The neighborhood political and cultural shifts occurring because of gentrification can lead to remaining residents losing their power to advocate for resources [43–45]. This may instigate feelings of resentment towards newcomers, [45] who may be threatened with declining relative income and displacement [22]. Thus, we had hypothesized that gentrification would result in improvements in perceptions of social cohesion and neighborhood solidarity amongst gentrifiers themselves [22, 31], while simultaneously reducing these perceptions in poorer residents remaining in the neighborhood. In contrast, our results suggest that the perceptions of collective efficacy of long-time residents of gentrified neighborhoods was not significantly different from perceptions of collective efficacy of gentrifiers, although a negative tendency was observed. However, newcomers to gentrifying neighborhoods did have higher perceived collective efficacy than individuals living in neighborhoods that did not gentrify.

We also conducted some exploratory analyses to see if gentrification might be related to health outcomes in our sample. Gentrification was not associated with self-perceived mental or physical health. This contrasts with previous research conducted with the Pennsylvania Household Health Survey that found that gentrification resulted in slightly higher self-rated health [41]. With respect to life-satisfaction, our findings indicated that individuals from gentrifying neighborhoods were more likely to be satisfied with life. We hypothesize that these feelings of life satisfaction could be linked to their positive feelings about the upgrades involved in the revitalization of their gentrified neighborhood.

### Strengths and limitations

These results do not suggest that all types of residents from gentrifying neighborhoods will benefit from gentrification. Gentrification is often associated with the displacement of the original inhabitants of the neighborhood [15, 27, 45, 46] an especially critical issue for renters living in gentrifying neighborhoods, who are more likely to have to make involuntary moves [47]. The cross-sectional nature of our study and the long time-period used to measure gentrification suggests that we may not have captured the perceptions of neighborhood collective efficacy of many vulnerable individuals that were displaced by gentrification. Rather, our survey likely captured the perceptions of neighborhood collective efficacy of many of the residents that had the financial resources to resist displacement. We distinguished the effect of gentrification on the collective efficacy of gentrifiers from the effect of gentrification on the collective efficacy of the original residents of gentrifying neighborhoods by creating a variable delineating if individuals had moved to their neighborhood in 1996 or later. However, although we can identify the neighborhoods in which gentrification occurred between 1996 and 2006, we had no way of knowing the exact year that gentrification began in each individual neighborhood. Strengths of this study include that the analyses from this paper were conducted with data from the first wave of a longitudinal cohort study making follow-up longitudinal analyses possible. This study is also the first to our knowledge to empirically test the relationship between gentrification and perceived collective efficacy.

## Conclusions

The positive relationship that was found between gentrification and perceived collective efficacy contradicts the notion that gentrification will necessarily create an environment in which residents are less trusting of their neighbors, and foster less political and social solidarity. This study responds to a gap in the literature by conducting an empirical investigation of the relationship between gentrification and collective efficacy. We suggest that future research explore this relationship in other cities and geographical areas.

## Footnotes

1 ECA-MSW is noteworthy for McGill’s Community-University Research Alliance Making Megaprojects Work for Communities because it contains the site and environment of the proposed McGill University Health Centre, which is the Alliance’s primary research focus.
